# Alterações da ECA2 e Fatores de Risco para Gravidade da COVID-19 em Pacientes com Idade Avançada

**DOI:** 10.36660/abc.20200487

**Published:** 2020-10-13

**Authors:** Caio de Assis Moura Tavares, Thiago Junqueira Avelino-Silva, Gil Benard, Francisco Akira Malta Cardozo, Juliana Ruiz Fernandes, Adriana Castello Costa Girardi, Wilson Jacob

**Affiliations:** 1 Universidade de São Paulo Faculdade de Medicina Hospital das Clínicas São PauloSP Brasil Instituto do Coração (InCor), Hospital das Clínicas, Faculdade de Medicina da Universidade de São Paulo (USP), São Paulo, SP - Brasil; 2 Faculdade Israelita de Ciências da Saúde Albert Einstein Hospital Israelita Albert Einstein São PauloSP Brasil Hospital Israelita Albert Einstein, Faculdade Israelita de Ciências da Saúde Albert Einstein, São Paulo, SP - Brasil; 3 Universidade de São Paulo Faculdade de Medicina Hospital das Clínicas São PauloSP Brasil Laboratório de Investigação Médica em Envelhecimento (LIM-66), Serviço de Geriatria, Hospital das Clínicas, Faculdade de Medicina, Universidade de São Paulo (USP), São Paulo, SP, Brasil; 4 Universidade de São Paulo Faculdade de Medicina Divisão de Clínica Dermatológica São PauloSP Brasil Laboratório de Investigação Médica LIM-56, Divisão de Clínica Dermatológica Faculdade de Medicina da Universidade de São Paulo (USP), São Paulo, SP – Brasil; 5 Universidade de São Paulo Instituto de Medicina Tropical de São Paulo São PauloSP Brasil Instituto de Medicina Tropical de São Paulo, Universidade de São Paulo (USP), São Paulo, SP - Brasil

**Keywords:** Envelhecimento, COVID-19, Imunossenescência, Fragilidade, Multimorbidade

O entendimento da relação entre envelhecimento e gravidade da COVID-19 é fundamental sob diversos aspectos: para o manejo clínico de pacientes com infecção pelo SARS-CoV-2, formação de políticas em saúde e para o reposicionamento de fármacos e/ou desenvolvimento de possíveis alvos terapêuticos destinados a esta população. Com ênfase nos mecanismos moleculares e fisiopatológicos subjacentes, discorreremos sobre os potenciais fatores de risco que podem contribuir para a gravidade da COVID-19 em pacientes com idade avançada: alterações da ECA2 (enzima conversora da angiotensina 2), imunossenescência e *inflammaging,* e presença de multimorbidade e fragilidade.

## ECA2, Infecção por SARS-CoV-2 e Envelhecimento

Alterações induzidas pelo envelhecimento em vias metabólicas podem, em parte, explicar a maior taxa de morbimortalidade por COVID-19 em pacientes idosos. Dentre estas, destacam-se aquelas pertencentes ao sistema renina-angiotensina (SRA), haja vista o papel crucial que esse sistema desempenha tanto na transmissibilidade viral^1^ como na patogenia da lesão pulmonar aguda e de sua forma mais grave, a síndrome da angústia respiratória aguda (SARA).^2^

Sabe-se que a ECA2 age como um receptor para a *proteína* estrutural *S* (*espícula*) do SARS-CoV-2,^1^ através da qual o vírus ganha acesso à célula hospedeira. Esse mecanismo envolve a interação da proteína S viral com o domínio extracelular da ECA2, desencadeando mudanças conformacionais que desestabilizam a membrana celular, propiciando a internalização do SARS-CoV-2 e da ECA2, a replicação viral, e a transmissão célula a célula.^1^^,^^3^

**Figura 1:**
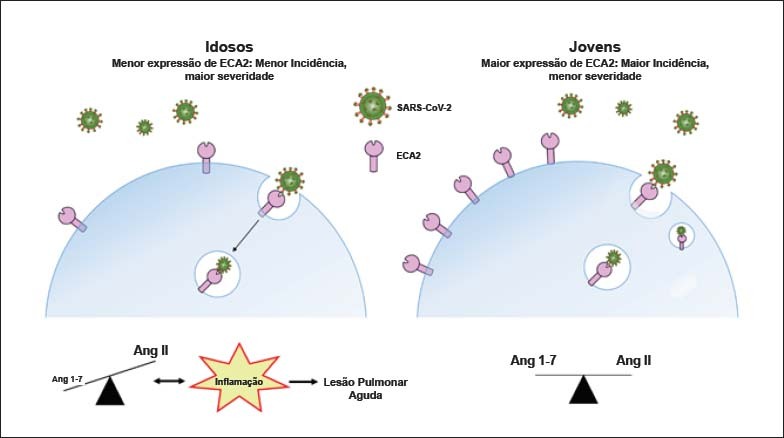
Associação entre idade, expressão de ECA2 e gravidade da COVID-19. A redução da expressão da ECA2 na membrana das células epiteliais pulmonares (pneumócitos tipo II), com o envelhecimento, aumenta os níveis de angiotensina II (Ang II) em detrimento da formação de Ang-(1-7), deflagrando, exageradamente, vias pró-inflamatórias, e predispondo pacientes idosos a maior gravidade de lesão pulmonar aguda e de mortalidade por COVID-19. Tal predisposição é potencializada pelo fato de que a ligação do SARS-CoV-2 a ECA2 leva à internalização de ambos, reduzindo ainda mais a expressão desta enzima na membrana celular. Em pacientes jovens, a expressão da ECA2 na membrana celular é maior do que em idosos, possibilitando a manutenção do equilíbrio entre as ações da Ang II e da Ang-1-7. A maior expressão de ECA2 pode causar aumento da infectividade por SARS-CoV-2, mas a geração de Ang-1-7 deflagra efeitos anti-inflamatórios que se contrapõem aos da Ang II, protegendo os indivíduos jovens contra o desenvolvimento/progressão da lesão pulmonar aguda. Este modelo é hipotético e não foi validado experimentalmente.

*Com o envelhecimento,* há redução considerável da expressão da ECA2 nos pulmões.^4^ Sabendo que a ECA2 constitui a porta de entrada para o SARS-CoV-2, é plausível postular que quanto maior a expressão da ECA2 na membrana celular, maior a infectividade. Todavia, apesar do decaimento da expressão da ECA2 tecidual com a idade, pacientes idosos apresentam maior gravidade de danos pulmonares e maior taxa de letalidade por COVID-19, quando comparados com indivíduos jovens.^5^ Uma hipótese proposta para explicar essa aparente incoerência entre idade avançada, nível de ECA2 tecidual e gravidade de infecção pelo SARS-CoV-2^6^ é a de que pessoas mais jovens, com maior expressão de ECA2, sejam as mais predispostas a terem a infecção, ao passo que indivíduos idosos, com menor expressão de ECA2, possam apresentar quadros mais graves ao serem infectados, devido à exacerbação dos efeitos mediados pela angiotensina II (Ang II). Essa hipótese é respaldada pelo fato de que, com o envelhecimento ocorra, além da redução da expressão de ECA2 tecidual, maior ativação de vias de sinalização pró-inflamatórias decorrentes da hiperatividade da via ECA/Ang II.^7^^-^^9^ Ademais, alinham-se a essa hipótese evidências amplas do papel protetor conferido pela ECA2 contra a insuficiência pulmonar e a existência de relação causal entre a via da ECA/Ang II e SARA, estabelecida em modelos animais.^10^^,^^11^ A complexa inter-relação entre ECA2, infecção por SARS-CoV-2 e envelhecimento está ilustrada na Figura 1.

## Imunossenescência, *Inflammaging* e COVID-19

Alterações significativas no sistema imune, que afetam tanto a imunidade inata quanto a imunidade adaptativa, têm sido associadas ao envelhecimento. Esse conjunto de alterações são agrupadas em um termo amplo denominado imunossenescência, caracterizado pelo declínio na responsividade do sistema imune, levando a desfechos mais graves de infecções virais e bacterianas, e aumento da incidência de doenças autoimunes, neoplasias, entre outras.^12^

**Figura 2:**
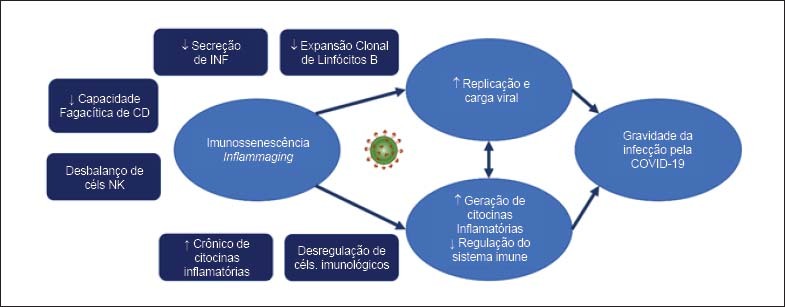
Relação entre imunossenescência, inflammaging e gravidade de infecção pela COVID-19. Note que as alterações do sistema imune e o estado inflamatório podem contribuir para a gravidade da infecção tanto por impactarem a replicação viral como pelo aumento de citocinas inflamatórias.

Com base no conhecimento atual das alterações causadas pela senescência no sistema imune, assim como pelos estudos publicados relacionados à fisiopatologia da COVID-19, é possível levantar hipóteses para explicar a elevada frequência de casos graves em idosos ou pacientes com doenças crônicas. Indivíduos hígidos em geral, ao contrário de idosos e imunodeprimidos, apresentam eficiente imunidade inata que, associada à imunidade celular e humoral intactas, permitem limitar a progressão da infecção e recuperação em algumas semanas. Essa resposta imune controlada supostamente atua na fase inicial do processo infeccioso, limitando replicação e disseminação viral, eventos que, infelizmente, ocorrem frequentemente nos idosos em estado crítico pela COVID-19.^13^^,^^14^ Em idosos, os sistemas imune inato e adaptativo, enfraquecidos, permitiriam cargas virais mais elevadas e persistentes — hipótese que está de acordo com descrição recente de pacientes com COVID-19, em que a carga viral detectada na orofaringe posterior se correlaciona com a idade.^15^ Essa carga viral aumentada representa estímulo antigênico intenso e persistente nos idosos, concomitantemente a uma menor regulação do sistema imune. A relação entre alterações no sistema imune, idade avançada e gravidade da COVID-19 está ilustrada na Figura 2. Para sua melhor compreensão, faz-se necessário definir e descrever os processos de imunossenescência e *inflammaging*.

As células dendríticas (CD) são células do sistema imune inato que fazem a conexão entre os sistemas imunológicos inato e adaptativo^16^ — esse subtipo de célula aparenta ser importante na defesa do hospedeiro contra o SARS-CoV-2, dada a localização deste tipo celular (presente na pele, cavidade nasal, pulmões, sangue periférico, sítios nos quais podemos encontrar o vírus). Em idosos, as CDs: 1) apresentam menor capacidade fagocítica — podendo resultar não somente em menor eficiência de resposta do sistema imune, mas também na redução da capacidade fisiológica de remover componentes próprios (incluindo células apoptóticas); 2) ficam menos capazes de instruir uma resposta imune adaptativa através de sinalização coordenada; 3) permanecem capazes de secretar citocinas inflamatórias sob condições de estresse*,* contribuindo para um estado inflamatório crônico.^17^^-^^19^

Além das CDs, monócitos e macrofágos atuam na imunidade inata através da produção de citocinas pró-inflamatórias e pelo processamento e apresentação de antígenos para linfócitos T. Com o envelhecimento, pode haver uma redução tanto na geração de precursores de macrófagos como na sua função fagocítica.^20^ Também já foi descrita a desregulação da sinalização por receptores do tipo *Toll* (*toll-like receptors* — TLR), gerando produção insuficiente de fator de necrose tumoral-*α* (*tumor necrosis factor-alpha* — TNF-*α*) e interleucina 6 (IL-6), e contribuindo para menor ativação de células imunes essenciais na resposta imunológica, como os linfócitos.^21^

A senescência causa, também, um desequilíbrio nas populações de células *Natural Killer* (NK) – que tem participação precoce na resposta imune a processos infecciosos e contribuem para orquestrar os passos subsequentes da resposta imune adaptativa. Há um aumento do subtipo CD56*dim* (alta capacidade citotóxica) e diminuição do subtipo CD56*bright* (baixa capacidade citotóxica, mas com elevada atividade imunorregulatória por meio de secreção de citocinas e quimiocinas), impactando tanto a resposta imune adaptativa quanto a capacidade de regulação do sistema imune.^22^^-^^25^

Além das alterações na imunidade inata, o envelhecimento impacta diretamente a resposta imune adaptativa mediada por linfócitos T e B. Idosos sabidamente apresentam produção atenuada de anticorpos e resposta vacinal reduzida. Diversos mecanismos contribuem para essa deficiência: 1) o balanço entre diferentes subtipos de linfócitos B é alterado, com maior proporção de células B de memória, que produzem grandes quantidades de citocinas inflamatórias e que contribuem para o status de inflamação sistêmica nesta população (estas células possivelmente tem um papel na geração e manutenção da inflamação sistêmica, conhecida como *inflammaging*);^26^ 2) Linfócitos B *naïve* de idosos são capazes de produzir IL-10 e TNF-*α* frente a um estímulo fisiológico, enquanto linfócitos B *naïve* de indivíduos jovens requerem estímulos mais potentes; 3) plasmócitos apresentam redução de sua expansão clonal com o envelhecimento, culminando na produção de anticorpos com menor afinidade antigênica;^27^^,^^28^ 4) a diminuição do repertório de linfócitos B causada pelo aumento do compartimento de células de memória impacta a capacidade de responder a novos estímulos antigênicos. A redução na capacidade da resposta humoral primária, por sua vez, associa-se a perturbações tanto no *switch* de classe de imunoglobulinas quanto na geração de anticorpos específicos com diferentes funções. Decorre, portanto, maior susceptibilidade a infecções.^29^

Em pacientes acometidos pela forma grave da COVID-19, o dano presente no parênquima pulmonar seria causado principalmente pela resposta inflamatória grave e menos pela ação direta do vírus. A resposta imune exacerbada (ou imunopatogênica) é responsável, ao menos em parte, pela pneumonia grave, insuficiência respiratória destes indivíduos, e eventualmente pelas alterações em outros órgãos e sistemas. Um dos problemas relacionados ao envelhecimento é que alguns idosos ou pacientes com doenças crônicas são incapazes de modular a resposta imune inflamatória, levando a um transbordamento de células do sistema imunológico e de citocinas inflamatórias nos pulmões, um evento denominado “*cytokine storm*” (tempestade de citocinas).^13^ Diversas das citocinas descritas no contexto de *inflammaging* como IL-6, TNF-*α* e interferon gama (IFN-*γ*) participam desta tempestade de citocinas. IL-6 também se relaciona à fragilidade, perda de massa muscular, declínio cognitivo e risco de hospitalização por pneumonia, manifestações frequentes em idosos frágeis. Esta citocina é desencadeadora de inflamação e injúria tecidual, que pode por sua vez facilitar a invasão de patógenos.^30^^,^^31^ Idosos apresentam níveis elevados de TNF-*α* após estimulação com lipopolissacarídeos (LPS) e IFN-*γ*.^32^ Esta citocina atua reduzindo a expressão de CD28 através da inibição de sua transcrição (no qual os mecanismos moleculares ainda não são bem conhecidos).^32^^-^^34^ Em pacientes com COVID-19, reportou-se aumento dos níveis de TNF-*α*, IL-6, IFN-*γ* e IL-10, ao passo que redução dos níveis destas citocinas se relacionou à resolução da doença.^35^ Achados semelhantes foram descritos por Huang e colegas, porém um painel ainda maior de citocinas inflamatórias foi analisado e incluiu interleucina 1 beta (IL-1*β*), IL-12 e proteína de quimioatração de monócitos 1 (MCP1).^36^

## Multimorbidade

A interação indivíduo-multimorbidade é complexa e interfere no gerenciamento clínico de pacientes com COVID-19. Se no seguimento rotineiro de pessoas com multimorbidade já devem ser consideradas as interações doença-doença, doença-tratamento, e tratamento-tratamento, no contexto da infecção pelo novo coronavírus é introduzida uma variável adicional,^37^ frequentemente acompanhada de novas disfunções orgânicas e efeitos pouco compreendidos.

**Figura 3:**
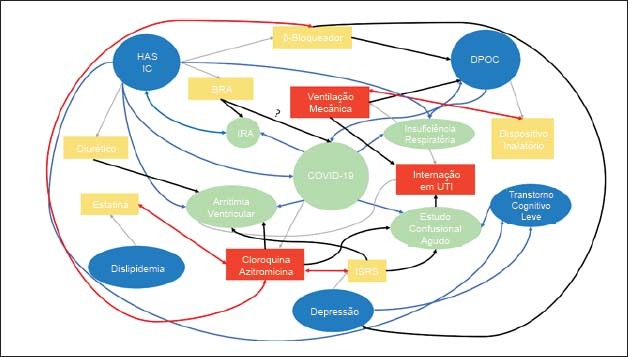
Modelo teórico de paciente com multimorbidade com COVID-19. Balões azuis representam doenças crônicas; balões verdes, complicações da COVID-19; retângulos amarelos tratamento para doenças crônicas; e retângulos vermelhos terapêutica nova, iniciada na internação. Linhas cinzas representam tratamento usual com base na doença; linhas azuis, possíveis interações entre as doenças; linhas vermelhas, possíveis interações entre os tratamentos propostos; e linhas pretas, possíveis interações entre o tratamento proposto para duas doenças diferentes. DPOC: doença pulmonar obstrutiva crônica; HAS: hipertensão arterial sistêmica; IC: insuficiência cardíaca; IRA: insuficiência renal aguda; ISRS: inibidor seletivo da recaptação de serotonina; UTI: unidade de terapia intensiva. Adaptado de.^37^^,^^38^

Em um exemplo teórico, pode-se imaginar um paciente de 72 anos com HAS, insuficiência cardíaca de fração de ejeção reduzida, doença pulmonar obstrutiva crônica (DPOC), dislipidemia, depressão e transtorno cognitivo leve em uso de estatina, bloqueador do receptor tipo 1 de angiotensina II (BRA), betabloqueador, dispositivo inalatório com beta-2-agonista de longa duração/corticoide inalatório e inibidor seletivo da recaptação de serotonina (ISRS). O paciente, ao ser hospitalizado com COVID-19, evolui com insuficiência respiratória aguda hipoxêmica e necessidade de internação em Unidade de Terapia Intensiva (UTI), com suporte de ventilação mecânica. São iniciadas cloroquina e azitromicina e, ao longo da internação, o paciente apresenta estado confusional agudo, insuficiência renal aguda e arritmia ventricular. A Figura 3 exemplifica as diversas possíveis interações neste cenário: interação entre doenças (linhas azuis), interação entre o tratamento para uma doença impactando em outra doença (linhas pretas) e interações entre os tratamentos (linhas vermelhas). A complexidade do cenário ilustra a importância do cuidado centrado no paciente para definição do plano terapêutico, dado que interações entre doenças tratamentos podem ser deletérias ao doente com multimorbidade.^37^^,^^38^

Apesar das informações disponíveis sugerirem uma associação entre multimorbidade e gravidade da COVID-19, não é claro ainda se existem situações específicas em que o tratamento de doenças crônicas possa ter um efeito benéfico no controle da infecção (ex.: HIV em uso de antirretroviral, fibrilação atrial permanente em uso de anticoagulação oral). Assim, enquanto não houver mais informações sobre a infecção pelo SARS-CoV-2, deve-se ter cuidado antes de suspender intempestivamente medicamentos aos quais o paciente está habituado. Cabe à equipe assistencial ponderar caso a caso os riscos e benefícios de cada medicamento em uso frente às possíveis intervenções contra a COVID-19. Evita-se, assim, a indesejada cascata em que a doença número 1 (COVID-19) seja controlada hoje, apenas para que as doenças número 2, 3, 4… (doenças crônicas) se tornem problemas ainda maiores amanhã.^37^^-^^39^

## Fragilidade

Apesar de todo idoso ter algum risco de desenvolver fragilidade, ela é mais comum entre aqueles que sofrem de multimorbidades, sedentarismo e alimentação inadequada.^40^ A fragilidade está associada a um estado pró-inflamatório crônico, caracterizado pelo aumento de citocinas como a interleucina 6 (IL-6) e fator de necrose tumoral alfa (TNF-*α*), cujos níveis podem predizer perda de funcionalidade e outros desfechos adversos em saúde.^41^ Levando essas informações em consideração, e sabendo que já existem resultados sugerindo a associação entre níveis elevados de IL-6 e maior mortalidade em indivíduos com COVID-19,^42^ é possível que a fragilidade seja um marcador prognóstico mais robusto do que idade, na doença.

Infelizmente, no cenário de uma pandemia, durante a qual decisões clínicas e alocações de recursos limitados precisam ser realizadas rapidamente, é comum que critérios puramente etários sejam empregados para definir os melhores candidatos para determinadas condutas.^43^ No entanto, é essencial entender que a população geriátrica é muito mais heterogênea que outros grupos etários, e que, assim, não é possível determinar uma correlação automática entre idade e o potencial para benefícios de um tratamento.^43^ Essa ressalva é particularmente verdadeira quando, por tratamento, entendem-se medidas de suporte clínico gerais e assistência médico-hospitalar adequada, como no caso da COVID-19.

Por outro lado, fragilidade ao classificar pessoas da mesma faixa etária, em diferentes níveis de risco para desfechos adversos, pode auxiliar na avaliação prognóstica de infectados pela COVID-19. Sua identificação é possível através de escalas simples, como a escala FRAIL e o Índice de Fragilidade, instrumentos de rastreio já validados para preenchimento com informações obtidas com pacientes, familiares ou dados de prontuário, flexibilização interessante em um cenário em que medidas de isolamento são necessárias.^44^^,^^45^

A identificação da fragilidade já a partir da unidade de pronto atendimento pode auxiliar na compreensão da doença aguda no contexto das condições basais de saúde do indivíduo, instrumentando a equipe para predição de eventos adversos. Com isso, é possível implementar intervenções voltadas à prevenção desses eventos adversos e guiar as decisões sobre disposição de recursos.^46^ Tal trabalho se integra à avaliação global do idoso, base fundamental da atuação de equipes multidisciplinares no gerenciamento de medicamentos, prevenção de quedas e delirium, e implementação de transições de cuidado.

A síndrome de fragilidade ainda não foi suficientemente estudada no contexto da COVID-19. Explorar sua utilidade para avaliação de prognóstico e definição da proporcionalidade de medidas de suporte pode ser passo fundamental para que profissionais da saúde possam agir com justiça e segurança, sem omissão ou negligência na aplicação dos recursos em saúde.

## Perspectivas

Está claro que os idosos serão aqueles mais impactados em morbimortalidade e, atuam aqui diferentes processos discutidos nesse artigo - ora conectados ora atuando sinergicamente: as alterações da ECA2 e do sistema renina-angiotensina, imunossenescência, *inflammaging*, multimorbidade e fragilidade, resumidas na Figura 3.

O SARS-CoV-2 impõe uma série de desafios aos gestores do sistema de saúde, governantes, profissionais de saúde e a sociedade em geral. Em um cenário com recursos finitos e saturação dos serviços de saúde, uma alocação racional do sistema de saúde se fará necessária.^47^ A tomada de decisão, no entanto, nunca deverá ser baseada única e exclusivamente na idade cronológica de um indivíduo — profissionais de saúde realocados para atendimento de pacientes com COVID-19 devem estar familiarizados com aplicação de *scores* de fragilidade determinados pela sua prática institucional e englobar parte do seu treinamento obrigatório. Cardiologistas sabem o impacto da fragilidade no tratamento de doenças cardiovasculares.^48^^,^^49^

O conhecimento gerado durante essa pandemia pode ser fundamental para fornecer respostas sobre peculiaridades do envelhecimento em diversos outros contextos. Possivelmente, o engajamento humano, tecnológico e da comunidade científica seja o maior de nossa história e que este recurso ímpar permita a implementação de novas terapêuticas, vacinas, amplie nossa capacidade diagnóstica com impacto inestimável na saúde dos idosos tanto para a COVID-19 como para outras doenças relacionadas ao envelhecimento.^50^
