# Supplementary material for: Alterações da ECA2 e Fatores de Risco para Gravidade da COVID-19 em Pacientes com Idade Avançada
Source: Arq Bras Cardiol. 2020 Oct 13;115(4):701–7. [Article in Portuguese] doi: 10.36660/abc.20200487 (PMC8386971; doi:10.36660/abc.20200487)
Supplement: Translation

# Translation: ACE2 Expression and Risk Factors for COVID-19 Severity in Patients with Advanced Age

**Authors:** Caio de Assis Moura Tavares, Thiago Junqueira Avelino-Silva, Gil Benard, Francisco Akira Malta Cardozo, Juliana Ruiz Fernandes, Adriana Castello Costa Girardi, Wilson Jacob

**Affiliations:** 1 São PauloSP Brazil Instituto do Coração (InCor), Hospital das Clínicas, Faculdade de Medicina da Universidade de São Paulo (USP), São Paulo, SP - Brazil; 2 São PauloSP Brazil Hospital Israelita Albert Einstein, Faculdade Israelita de Ciências da Saúde Albert Einstein, São Paulo, SP - Brazil; 3 São PauloSP Brazil Laboratório de Investigação Médica em Envelhecimento (LIM-66), Serviço de Geriatria, Hospital das Clínicas, Faculdade de Medicina, Universidade de São Paulo (USP), São Paulo, SP, Brazil; 4 São PauloSP Brazil Laboratório de Investigação Médica LIM-56, Divisão de Clínica Dermatológica Faculdade de Medicina da Universidade de São Paulo (USP), São Paulo, SP – Brazil; 5 São PauloSP Brazil Instituto de Medicina Tropical de São Paulo, Universidade de São Paulo (USP), São Paulo, SP - Brazil

**Keywords:** Aging, COVID-19, Immunosenescence, Frailty, Multimorbidity

Understanding the relationship between aging and COVID-19 severity is key from multiple perspectives: for the clinical management of patients with SARS-CoV-2 infection, drafting of health policies and repositioning of drugs and/or the development of potential therapeutic targets for this population. Focusing on the underlying molecular and pathophysiological mechanisms, we will discuss the potential risk factors that may contribute to COVID-19 severity in patients with advanced age: ACE2 (angiotensin-converting enzyme 2) expression, immunosenescence/inflammaging and the presence of multimorbidity or frailty.

## ACE2, SARS-CoV-2 Infection and Aging

Abnormalities induced by aging in metabolic pathways may partly explain the higher rate of COVID-19 morbidity and mortality in elderly patients. These include those belonging to the renin-angiotensin system (RAS), given the crucial role that this system plays both in viral transmissibility^1^ and in the pathogenesis of acute lung injury and its most severe form: acute respiratory distress syndrome (ARDS).^2^

ACE2 is known to act as a receptor for SARS-CoV-2 1 structural protein S (spike),^1^ through which the virus gains access to the host cell. This mechanism involves interaction of viral S protein with ACE2 extracellular domain, triggering conformational changes that destabilize the cell membrane, allowing the internalization of SARS-CoV-2 and ACE2, viral replication, and cell-to-cell transmission.^1^^,^^3^

With aging, there is a considerable reduction in the expression of ACE2 in the lungs.^4^ Knowing that ACE2 is the gateway to SARS-CoV-2, it can be affirmed that the greater the expression of ACE2 in the cell membrane, the greater the infectivity. However, despite the decline in tissue ACE2 expression with age, elderly patients have greater severity of lung damage and higher lethality rate from COVID-19 compared to young individuals.^5^ A possible explanation to this apparent inconsistency between old age, level of tissue ACE2 and severity of SARS-CoV-2 infection^6^ is that younger people with higher ACE2 expression are more likely to have the infection while elderly individuals, with lower ACE2 expression may present more severe conditions when infected due to exacerbated effects mediated by Angiotensin II (Ang II). This is supported by the fact that, with aging, in addition to reduced tissue ACE2 expression, there is greater activation of pro-inflammatory signaling pathways resulting from hyperactivity of the ACE/Ang II pathway.^7^^-^^9^ Also, there is ample evidence of the protective role of ACE2 against pulmonary insufficiency and a causal relationship between the ACE/Ang II pathway and ARDS, established in animal models.^10^^,^^11^ The complex interrelationship between ACE2, SARS-CoV-2 infection and aging is illustrated in Figure 1.

**Figure 1:** Association between age, ACE2 expression and COVID-19 severity. Reduced expression of ACE2 in the membrane of pulmonary epithelial cells (type II pneumocytes) with aging increases the levels of angiotensin II (Ang II) to the detriment of Ang-(1-7) formation, exaggeratedly triggering pro-inflammatory pathways and predisposing elderly patients to greater severity of acute lung injury and mortality from COVID-19. Such predisposition is enhanced by the fact that SARS-CoV-2 binding to ACE2 leads to the internalization of both, further reducing the expression of this enzyme in the cell membrane. In young patients, expression of ACE2 in the cell membrane is greater than in the elderly, enabling a balance between the actions of Ang II and Ang-1-7. Greater expression of ACE2 may cause increased infectivity by SARS-CoV-2, but the generation of Ang-1-7 triggers anti-inflammatory effects that are opposed to those of Ang II, protecting young individuals against the development/progression of acute lung injury. This model is hypothetical and has not been validated experimentally.

## Immunosenescence, Inflammaging and COVID-19

Significant abnormalities in the immune system, which affect both innate and adaptive immunity, have been associated with aging. This set of abnormalities are broadly referred to as immunosenescence, characterized by a decline in immune system responsiveness, leading to more serious viral and bacterial infection outcomes, and increased incidence of autoimmune diseases, neoplasms, and others.^12^

Based on current knowledge of the abnormalities caused by immune system senescence, and based on studies related to the pathophysiology of COVID-19, it is possible to come up with explanations about the high frequency of severe cases in the elderly or patients with chronic diseases. Healthy individuals in general, in contrast to the elderly and immunosuppressed, have efficient innate immunity, which, associated with intact cellular and humoral immunity, limit the progression of infection and recovery in a few weeks. This controlled immune response supposedly acts in the initial phase of the infectious process, limiting viral replication and dissemination, which, unfortunately, frequently occur in the elderly critically ill from COVID-19.^13^^,^^14^ In the elderly, weakened innate and adaptive immune systems would allow higher and persistent viral loads — an assumption that is in line with a recent description of patients with COVID-19, in which the viral load detected in the posterior oropharynx correlates with age.^15^ This increased viral load represents intense and persistent antigenic stimulus in the elderly, concomitantly with lower immune system regulation. The relationship between changes in the immune system, advanced age and COVID-19 severity is shown in Figure 2. To better understand this, it is necessary to define and describe the processes of immunosenescence and inflammaging.

**Figure 2:** Relationship between immunosenescence, inflammageing and severity of infection by COVID-19. Note that changes in the immune system and inflammatory status can contribute to the severity of the infection both by impacting viral replication and by increasing inflammatory cytokines.

Dendritic cells (DC) are cells of the innate immune system that connect the innate and adaptive immune systems^16^ — this cell subtype seems important in the host’s defense against SARS-CoV-2, given the location of this cell type (present in the skin, nasal cavity, lungs, peripheral blood, the sites where we can find the virus). In the elderly, DCs: 1) have poorer phagocytic capacity — which may result not only in a less efficient immune system response, but also in reduced physiological capacity to remove proper components (including apoptotic cells); 2) are less able to instruct an adaptive immune response through coordinated signaling; 3) remain capable of secreting inflammatory cytokines under stress conditions, contributing to a chronic inflammatory state.^17^^-^^19^

In addition to DCs, monocytes and macrophages act on innate immunity by producing pro-inflammatory cytokines and processing and presenting antigens to T lymphocytes. With aging, there may be reduced generation of macrophage precursors and their phagocytic function.^20^ Signaling dysregulation by Toll-like receptors (TLR) has also been described, generating insufficient production of tumor necrosis factor-alpha (TNF-*α*) and interleukin-6 (IL-6), and contributing to poorer activation of essential immune cells in immune response, such as lymphocytes.^21^

Senescence also causes an imbalance in Natural Killer (NK) cell populations — which play an early role in the immune response to infectious processes and participate on orchestrating the subsequent steps of the adaptive immune response. There is an increase in the subtype CD56dim (high cytotoxic capacity) and a decrease in the subtype CD56bright (low cytotoxic capacity, but with high immunoregulatory activity through secretion of cytokines and chemokines) impacting both adaptive immune response and regulatory capacity of the immune system.^22^^-^^25^

In addition to changes in innate immunity, aging directly impacts the adaptive immune response mediated by T and B lymphocytes. Elderly people are known to have attenuated antibody production and reduced vaccine response. Several mechanisms contribute to this deficiency: 1) the balance between different subtypes of B lymphocytes is altered, with a higher proportion of memory B cells, which produce large amounts of inflammatory cytokines and contribute to the status of systemic inflammation in this population (these cells possibly have a role in the generation and maintenance of systemic inflammation, known as inflammaging);^26^ 2) Naïve B lymphocytes in the elderly are capable of producing IL-10 and TNF-*α* with physiological stimulus, while naïve B lymphocytes in young individuals require more potent stimuli; 3) plasmocytes have reduced clonal expansion with aging, culminating in the production of antibodies with lesser antigenic affinity;^27^^,^^28^ 4) decreased B lymphocyte repertoire caused by increased memory cell compartment impacts the ability to respond to new antigenic stimuli. Reduced primary humoral response capacity, in turn, is associated with disturbances both in the switch of immunoglobulin class and in the generation of specific antibodies with different functions. Therefore, there is greater susceptibility to infections.^29^

In patients affected by the severe form of COVID-19, lung parenchyma damage would be caused mainly by severe inflammatory response and less by the direct virus action. Exacerbated immune (or immunopathogenic) response partly accounts for severe pneumonia, respiratory failure in these individuals, and often for disorders in other organs and systems. One of the problems related to aging is that some elderly people or patients with chronic diseases are unable to modulate inflammatory immune response, leading to an overflow of immune cells and inflammatory cytokines in the lungs, an event called “cytokine storm”.^13^ Several of the cytokines described in the context of inflammaging, such as IL-6, TNF-α and interferon gamma (IFN-γ) participate in this storm of cytokines. IL-6 is also related to frailty, loss of muscle mass, cognitive decline and risk of hospitalization for pneumonia, frequent manifestations in frail elderly people. This cytokine triggers inflammation and tissue injury, which may facilitate the invasion of pathogens.^30^^,^^31^ Elderly people have high levels of TNF-α after stimulation with lipopolysaccharides (LPS) and IFN-γ.^32^ This cytokine reduces the expression of CD28 by inhibiting its transcription (in which the molecular mechanisms are not yet well known).^32^^-^^34^ In patients with COVID-19, increased levels of TNF-α, IL-6, IFN-γ and IL-10 have been reported; whereas reduced levels of these cytokines were related to the resolution of the disease.^35^ Similar findings were described by Huang et al., but an even larger panel of inflammatory cytokines was analyzed and included interleukin 1 beta (IL-1β), IL-12 and monocyte chemoattractant protein 1 (MCP1).^36^

## Multimorbidity

Individual-multimorbidity interaction is complex and interferes with the clinical management of patients with COVID-19. If in the routine follow-up of people with multimorbidity disease-disease, disease-treatment and treatment-treatment interactions should already be considered, in the context of infection with the novel coronavirus, an additional variable^37^ is introduced, often accompanied by new organic dysfunctions and poorly understood effects.

In a theoretical example, one can imagine a 72-year-old patient with Hypertension, heart failure with reduced ejection fraction, chronic obstructive pulmonary disease (COPD), dyslipidemia, depression and mild cognitive impairment using statins, angiotensin II type 1 receptor blocker (ARB), beta-blocker, inhalation device with long-acting beta-2-agonist/inhaled corticoid and selective serotonin reuptake inhibitor (SSRI). If this patients is hospitalized with COVID-19 presents acute hypoxemic respiratory failure and requires admission to the Intensive Care Unit (ICU) with mechanical ventilation. Chloroquine and azithromycin are initiated and, during hospitalization, the patient presents acute confusional state, acute kidney failure and ventricular arrhythmia. Figure 3 exemplifies the various possible interactions in this scenario (interaction between diseases (blue lines), interaction between treatment for one disease impacting another disease (black lines) and interactions between treatments (red lines). This complex scenario illustrates the importance of patient-centered care to define the therapeutic plan, given that interactions between diseases and treatments can be harmful to patients with multimorbidity.^37^^,^^38^

**Figure 3:** Theoretical model of a patient with multimorbidity with COVID-19. Blue balloons represent chronic diseases; green balloons, COVID-19 complications; yellow rectangles, treatment for chronic diseases; and red rectangles, new therapy initiated on admission. Gray lines represent usual treatment based on the disease; blue lines, potential disease interactions; red lines, potential interactions between the proposed treatments; and black lines, potential interactions between the proposed treatment for two different diseases. COPD: chronic obstructive pulmonary disease; HTN: Hypertension; HF: heart failure; AKI: Acute Kidney Injury; SSRI: selective serotonin reuptake inhibitor; ICU: intensive care unit. Adapted from.^37^^,^^38^

Although the available information suggests an association between multimorbidity and severity of COVID-19, it is still unclear whether there are specific situations in which the treatment of chronic diseases may have a beneficial effect on infection control (e.g.: HIV using antiretroviral, permanent atrial fibrillation using oral anticoagulation). Thus, while there is no more information about SARS-CoV-2 infection, care should be taken before untimely suspending medications to which the patient is accustomed. The healthcare team is responsible for weighing, on a case-by-case basis, the risks and benefits of each drug in use in view of possible interventions against COVID-19. This avoids the unwanted cascade in which disease number 1 (COVID-19) is controlled today and diseases number 2, 3, 4 ... (chronic diseases) become even bigger problems tomorrow.^37^^-^^39^

## Frailty

Although every elderly person has some risk of developing frailty, it is more common among those with multimorbidities, physical inactivity and inadequate nutrition.^40^ Frailty is associated with a chronic pro-inflammatory state characterized by increased cytokines such as interleukin 6 (IL-6) and tumor necrosis factor alpha (TNF-α), whose levels can predict loss of functionality and other adverse health outcomes.^41^ Taking this information into account, and knowing that there are outcomes suggesting an association between high levels of IL-6 and higher mortality in individuals with COVID-19,^42^ frailty is likely a more robust prognostic marker than age, in the disease.

Unfortunately in the context of a pandemic, during which clinical decisions and limited resources need to be arranged quickly, it is common for purely age criteria to be used to define the best candidates for certain managements.^43^ However, it is essential to understand that the geriatric population is much more heterogeneous than other age groups, and that, therefore, it is not possible to determine an automatic correlation between age and the potential for treatment benefits.^43^ This caveat is particularly true when treatment includes general clinical support measures and proper medical and hospital assistance, as in COVID-19.

On the other hand, classifying people of the same age group according to frailty, at different levels of risk for adverse outcomes, can assist in the prognostic assessment of those infected with COVID-19. Its identification is possible through simple scales, such as the FRAIL scale and the Frailty Index, as these screening instruments are currently validated for filling in information from patients, family members or medical records, an interesting flexibility in a scenario where isolation measures are necessary.^44^^,^^45^

The identification of frailty from the emergency care unit can help understand acute illness in the context of an individual’s baseline health conditions, hence helping the team to predict adverse events. Therewith, it is possible to implement interventions aimed at preventing these adverse events and guide the decisions on allocation of resources.^46^ Such work is part of the global assessment of the elderly, the cornerstone in the multidisciplinary teams’ work of managing medications, preventing falls and delirium, and implementing care transitions.

Frailty syndrome has not been sufficiently studied in the context of COVID-19. Exploring its usefulness for assessing prognosis and defining the proportionality of support measures can be a fundamental step so that health professionals may act with justice and security, without omission or negligence in the application of health resources.

## Prospects

It is clear that the elderly will be those who will be most impacted by the pandemic, regarding morbidity and mortality — this includes different aspects discussed in this study, connected in some aspects and acting synergistic in others: expression of ACE2 and renin-angiotensin system, immunosenescence, inflammaging, multimorbidity and frailty, summarized in Figure 3.

The SARS-CoV-2 imposes a range of challenges on health system managers, government officials, health professionals and society in general. In a scenario with finite resources and saturation of health services, a rational allocation of the health system will be necessary.^47^ Decision-making, however, should never be based solely and exclusively on an individual’s chronological age — healthcare professionals relocated to see patients with COVID-19 must be familiar with the application of frailty scores determined by their institutional practice and complete mandatory training. Cardiologists are aware of the impact of frailty in the treatment of cardiovascular diseases.^48^^,^^49^

The knowledge generated during this pandemic can be essential to provide answers about the peculiarities of aging in several other contexts. Human, technological and scientific community engagement is possibly the greatest in our history and this unique resource may allow the implementation of new therapies, vaccines, expand our diagnostic capacity with an inestimable impact on the health of the elderly, both for COVID-19 and for other diseases related to aging.^50^
